# Usefulness of urinary potassium to creatinine ratio to predict diuretic response in patients with acute heart failure and preserved ejection fraction

**DOI:** 10.1002/clc.24040

**Published:** 2023-06-07

**Authors:** Pau Llàcer, Julio Núñez, François Croset, Marina García, Martín Fabregate, Raúl Ruiz, Genoveva López, Cristina Fernández, Beatriz Del Hoyo, Jorge Campos, Antonio Gomis, Luis Manzano

**Affiliations:** ^1^ Internal Medicine Department Hospital Universitario Ramón y Cajal, IRYCIS Madrid Spain; ^2^ Department of Medicine and Medical Specialties, Facultad de Medicina y Ciencias de la Salud Universidad de Alcalá Madrid Spain; ^3^ Cardiology Department, Hospital Clínico Universitario Universitat de València, INCLIVA Valencia Spain; ^4^ CIBER Cardiovascular Madrid Spain; ^5^ Nephrology Department Hospital Universitario Ramón y Cajal Madrid Spain

**Keywords:** acute heart failure, diuretic response, preserved ejection fraction, urinary potassium creatinine ratio

## Abstract

**Background:**

Patients with acute heart failure (AHF) require intensification in the diuretic strategy. However, the optimal diuretic strategy remains unclear. In this work, we aimed to evaluate the role of urinary potassium to creatinine ratio (K/Cr) to predict diuretic and natriuretic response to thiazide or mineralocorticoid receptor antagonists (MRAs) in a cohort of patients with AHF and preserved ejection fraction (AHF‐pEF).

**Hypothesis:**

Patients with a high urinary K/Cr ratio will have a better diuretic and natriuretic response with spironolactone versus chlorthalidone.

**Methods:**

This is a study of 44 patients with AHF‐pEF with suboptimal loop diuretic response. The primary endpoint was the baseline K/Cr associated with natriuretic and diuretic effect of chlorthalidone versus spironolactone at 24 and 72 h. Mixed linear regression models were used to analyze the endpoints. Estimates were reported as least squares mean with their respective 95% confidence interval (CIs).

**Results:**

The median age of the study population was 85 years (82.5−88.5), and 30 (68.2%) were women. The inferential multivariate analysis suggested a greater natriuretic and diuretic effect of chlorthalidone across K/Cr levels. In the upper category, chlorthalidone translated into a statistically increase in natriuresis at 24 and 72 h. Chlorthalidone versus spironolactone showed ∆uNa of 25.7 mmol/L at 24 h (95% CI = −3.7 to 55.4, *p* = .098) and ∆uNa of 24.8 mmol/L at 72 h (95% CI = −4 to 53.6, *p* = .0106). The omnibus *p* value is .027. Multivariate analyses revealed a significant increase in 72 h cumulative diuresis irrespective of K/Cr status in those on chlorthalidone.

**Conclusions:**

In patients with AHF‐pEF and suboptimal diuretic response, diuresis and natriuresis are higher with the administration of chlorthalidone over spironolactone. These data don't support the hypothesis that the K/Cr ratio can help guide the choice of thiazide diuretic versus MRA in AHF‐pEF patients on loop diuretic.

## Graphical abstract

Prospective observational study evaluating the role of urinary potassium to creatinine ratio (K/Cr) to predict diuretic and natriuretic response to thiazide or mineralocorticoid receptor antagonists in a cohort of patients with acute heart failure (AHF) with preserved ejection fraction (AHF‐pEF) and suboptimal loop diuretic response. Diuresis and natriuresis are higher with the administration of chlorthalidone compared to spironolactone, irrespective of K/Cr status.

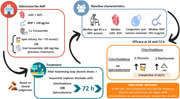

## 1 INTRODUCTION

Fluid overload or fluid redistribution is the main reason for hospital admission in acute heart failure (AHF), followed by increased cardiac filling pressures.^1^ The mainstay of treatment for AHF consists of the administration of intravenous (i.v.) loop diuretics, with or without the addition of other diuretics.^2^, ^3^ In most patients, administration of i.v. loop diuretics is sufficient to obtain clinical relief and mitigate signs of fluid overload. However, in some patients a sequential nephron blockage with additional diuretic is necessary.^4^, ^5^ The evidence supporting the efficacy and safety of the diuretic sequence in patients with suboptimal loop diuretic response is scarce. Indeed, the benefit of adding thiazides to loop diuretics in patients with AHF is based on small observational studies and not on randomized clinical trials.^2^, ^6^, ^7^ In addition, the diuretic effect of mineralocorticoid receptor antagonist (MRA) in patients with diuretic resistance is controversial.^8^, ^9^, ^10^, ^11^ We recently published that in patients with AHF treated with i.v. furosemide, the addition of chlorthalidone was associated with a short‐term greater natriuresis and urine output compared to addition of spironolactone.^12^ However, given that hyperaldosteronism is a key feature of diuretic resistance, and it translates into an increase in urine potassium levels, we hypothesize that high urine potassium will identify a subset of patients that benefits from MRA administration. Conversely, low urine potassium will identify those in which thiazides will provide a stronger natriuretic response.

In this work, we aimed to evaluate the role of urinary potassium to creatinine ratio (K/Cr) to predict diuretic and natriuretic response to thiazide or MRA in a cohort of patients with AHF with preserved ejection fraction (AHF‐pEF) and suboptimal loop diuretic response.

## 2 METHODS

### 2.1 Study population and protocol

This is a substudy of ICARPo, a prospective observational study of hospitalized patients with AHF‐pEF, treated with i.v. loop diuretics in which an additional diuretic (chlorthalidone or spironolactone) was prescribed.^12^

The study population included patients with AHF‐pEF admitted for AHF to the Internal Medicine Department of a third‐level teaching hospital center between June 2020 and March 2021. Patients meeting all of the following criteria were included: (1) older than 18 years; (2) episode of AHF requiring hospital admission and treatment with i.v. furosemide; (3) spot urinary sodium (uNa) <70 mmol/L in the first 2 h after i.v. furosemide administration or previous treatment with oral furosemide ≥80 mg/day at least 1 month before admission; (4) basal functional class NYHA II−IV; (5) findings (during index hospitalization or 180 days prior) of brain natriuretic peptide (BNP) >100 pg/mL, left ventricular ejection fraction (LVEF) >50%, and LV hypertrophy (septum or posterior wall >11 mm or E/e' >13); (6) capable of giving signed informed consent.

Patients with any following conditions were excluded: treatment with MRA and/or thiazides (in the previous month); estimated glomerular filtrate rate <30 mL/min/1.73 m^2^; serum K >5 mmol/L; recent acute coronary syndrome; systolic blood pressure (SBP) <90 mmHg; requirement of inotropes.

**Figure 1:**
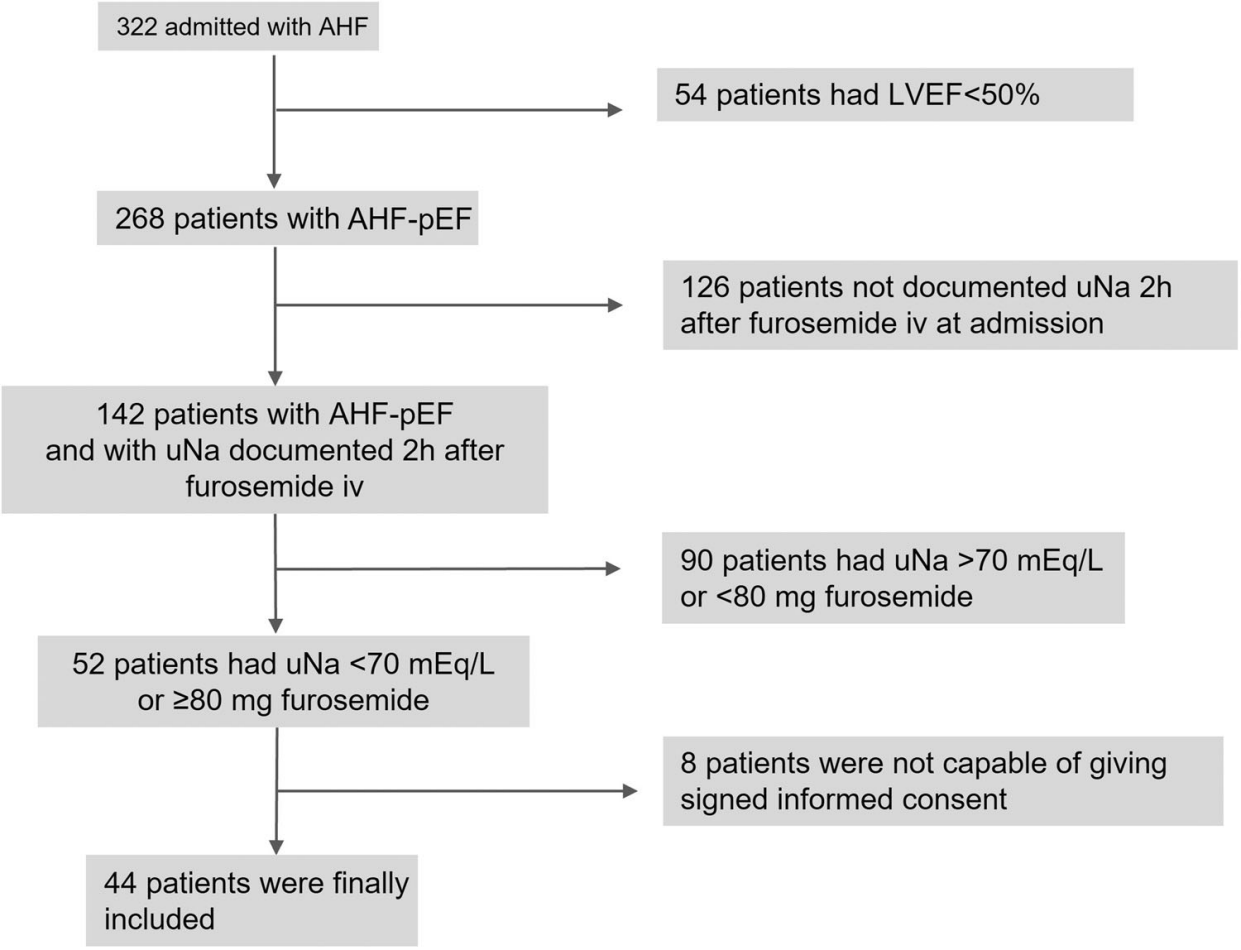
Flow chart of patients admitted with acute heart failure in the period under study. AHF‐pEF, AHF and preserved ejection fraction; LVEF, left ventricular ejection fraction; uNa, urinary sodium.

A total of 322 patients were admitted with the diagnosis of AHF in the period mentioned. Of these, 268 had preserved ejection fraction, and only 142 had uNa documented after 2 h furosemide i.v. Finally, 44 patients were included with spot uNa <70 mmol/L in the first 2 h after i.v. furosemide administration or ≥80 mg furosemide (Figure 1). Of the 44 patients included, 33 (75%) had uNa <70 mEq/L, 32 (72.7%) took 80 mg or more of furosemide, and 21 had both conditions (47.7%). Only 11 patients (25%) had the criterion of more than 80 mg furosemide with uNa over 70 mmol/L.

Baseline characteristics were recorded in preestablished electronic questionnaires including demographic data; a complete medical history, including current treatment and medications taken within the last 30 days; recording of vital signs; a complete physical examination; electrocardiogram; chest X‐ray; blood tests, including hematology and chemistry (sodium, potassium, parameters of renal function, carbohydrate antigen 125 [CA125],^13^ BNP); and urine electrolyte determination.

In‐hospital visits were performed at 24 h, 72 h, and 30 days according to usual clinical care. These visits included vital signs, complete physical examination, diuresis volume (24 and 72 h), and collection of blood and urine samples. During the follow‐up all concomitant medications and clinical adverse events (death from all causes or new worsening of AHF) were recorded.

To quantify natriuresis, a urine sample was taken at 8 a.m. at 24 and 72 h visits. To quantify 24 h diuresis, the patients collected it themselves in a container, but if accurate urinary collections were not deemed possible, a bladder catheter was placed.

#### 2.1.1 Diuretic strategy

The protocol of our institution advocates for an intensive diuretic treatment using a sequential nephron blockade (chlorthalidone or spironolactone) after maximizing loop diuretic doses. Chlorthalidone or spironolactone was prescribed the first 24 h following admission. The dose used for chlorthalidone was 25 mg (25−50) and for spironolactone 25 mg (25−50) in all cases during at least 72 h. The decision of the additional type of diuretic was based on clinical judgment.

### 2.2 Endpoint

The endpoint of interest was between treatment differences (chlorthalidone vs. spironolactone) in 24 and 72 h natriuresis and 72 h diuresis across K/Cr.

### 2.3 Statistical analysis

Continuous variables were presented as median and interquartile range (IQR), expressed as (Q_1_−Q_3_). Discrete data were expressed as frequency and percentages. The *χ* ^2^ test or Wilcoxon rank sum test was used as appropriate to compare baseline characteristics between the two treatment groups. K/Cr was categorized by the median of the distribution for exploratory purposes.

A mixed linear regression model was used for the analyses of changes in natriuresis. All analyses included adjustments for the treatment × visit interaction term (24 and 72 h) and the baseline value (before treatment) of the regression result. Estimates were reported as least squares means (LSM) with their respective 95% confidence intervals (CIs). The inclusion of other covariates was based on clinical judgment or inequalities in treatment groups at baseline. The covariates included in the final model were age, total furosemide dose, estimated glomerular filtration rate, K/Cr, and baseline uNa.

A multivariate linear regression model, adjusted for age, total furosemide dose, baseline uNa, and K/Cr, was used to analyze the 72 h cumulative urine output. The covariates contribution to the predictive ability of this multiple linear regression model was assessed by the coefficient of determination (*R* ^2^).

A two‐tailed *p*‐value of .05 was considered as significant for all analyses. The analyses were performed with Stata v15.1 (Stata Statistical Software).

## 3 RESULTS

### 3.1 Baseline characteristics

A total of 44 patients were included in the study. Spironolactone was added in 25 patients and chlorthalidone in 19 of them. The median age (IQR) of the study population was 85 years (82.5−88.5), and 30 (68.2%) were women. Table 1 shows the characteristics of the study cohort. Overall, most of the patients showed clinical, radiological, and biochemical signs of congestion. All patients had preserved LVEF, median (IQR) LVEF 65% (62.9−68.25). The median (IQR) of GFR, BNP, and uNa at admission were 50.7 mL/min/1.73 m^2^ (35.3−65.2), 491.6 pg/mL (285.2−732.6), and 45.5 mmol/L (24.5−76.5), respectively. There were no significant differences between treatment groups except for lower SBP in the spironolactone group (Table 1).

**Table 1:** Baseline characteristics.^a^

Variables	Total (*n* = 44)	Spironolactone (*n* = 25)	Chlortalidone (*n* = 19)	*p* Value
Age, years	85 (82.5−88.5)	85 (83−90)	85 (82−88)	.577
Women, *n* (%)	30 (68.2)	17 (68)	13 (68.4)	.976
Medical history				
Arterial hypertension, *n* (%)	40 (90.9)	24 (96.0)	16 (84.2)	.178
Diabetes mellitus, *n* (%)	23 (52.3)	13 (52.0)	10 (52.6)	.967
Chronic heart failure, *n* (%)	35 (79.5)	19 (76.0)	16 (84.2)	.504
Atrial fibrilation, *n* (%)	37 (84.1)	21 (84.0)	16 (84.2)	.985
Coronary artery disease, *n* (%)	14 (31.8)	7 (28.0)	7 (36.8)	.533
Chronic kidney disease, *n* (%)	26 (59.1)	14 (56.0)	12 (63.2)	.632
COPD, *n* (%)	7 (15.9)	4 (16.0)	3 (15.8)	.985
Chronic treatment				
ACE/ARB/ARNI, *n* (%)	28 (63.6)	16 (64.0)	12 (63.2)	.954
Beta‐blockers, *n* (%)	30 (68.2)	18 (72.0)	12 (63.2)	.533
Loop diuretics, *n* (%)	43 (97.7)	24 (96.0)	19 (100.0)	.378
Digoxin, *n* (%)	6 (13.6)	4 (16.0)	2 (10.5)	.600
Nitrates, *n* (%)	4 (9.0)	1 (4.0)	3 (15.8)	.178
Dihydropyridine calcium antagonists, *n* (%)	13 (29.5)	7 (28.0)	6 (31.6)	.797
Statins, *n* (%)	25 (56.8)	14 (56.0)	11 (57.9)	.900
Vital signs				
Heart rate, beats/min	74 (64−85)	76 (63−85)	74 (66−85)	.831
SBP, mmHg	130.5 (119.5−145.5)	126 (115−135)	141 (124−160)	.002
DBP, mmHg	72 (62−78)	70 (59−77)	73 (66−78)	.618
Clinical presentation				
Crackles, *n* (%)	41 (93.2)	22 (88)	19 (100)	.118
Edema, *n* (%)	43 (97.7)	25 (100)	18 (94.7)	.246
Ecocardiography				
LVEF, %	65 (62.9−68.25)	66.2 (65−70)	65 (62.9−65)	.082
LA volume index, mL/m^2^	47.8 (43−60)	47.5 (43−55.7)	50.2 (44−75.2)	.463
Septum, mm	11.5 (11−13.1)	11 (11−13)	12 (11−13.1)	.258
Chest X‐ray				
Pleural effusion, *n* (%)	42 (95.4)	23 (92)	19 (100)	.207
Laboratory				
Hemoglobine, g/dL	11.5 (10.1−12.7)	11.5 (10.5−12.5)	11.5 (9.8−13.4)	.767
Hematocrit, %	35.4 (31.8−40.3)	36.2 (33.3−39.4)	34.7 (31.4−40.6)	.434
Plasma sodium, mmol/L	140 (138−142)	140 (138−142)	140 (138−142)	.803
Plasma potasium, mmol/L	4.2 (3.8−4.5)	4 (3.7−4.5)	4.3 (3.9−4.9)	.226
Urea, mg/dL	66 (44.5−83.5)	69 (49−84)	65 (44−81)	.887
Creatinine, mg/dL	1.2 (0.9−1.5)	1.1 (0.9−1.5)	1.2 (0.8−1.5)	.803
BNP, pg/mL	491.6 (285.2−732.6)	505 (311.7−821.0)	485.3 (260.7−549.0)	.441
CA125, U/mL	69.4 (38.3−128.05)	112 (48.3−145.8)	60.3 (24.5−104.6)	.066
AST, U/L	19 (17−23.5)	19 (18−3)	18 (15−22)	.144
ALT, U/L	14.5 (11−20.5)	14 (12−21)	16 (9−19)	.280
eGFR mL/min/1.73 m^2^	50.75 (35.3−65.2)	47.82 (36.5−62.7)	51.2 (32.2−66.4)	.661
Urine sodium, mmol/L	45.5 (24.5−76.5)	45 (24−67)	46 (27−92)	.434
Urine potassium, mmol/L	30.1 (19.8−46.4)	30.6 (24.2−38.9)	28.8 (18.5−51.2)	.749
Urine creatinine, mmol/L	54 (23.6−81.2)	59.4 (27.9−81)	52 (23.3−95)	.557
Intravenous diuretic treatment administered				
72 h dose furosemide, mg	480 (340−560)	520 (380−600)	400 (340−500)	.096
Length of stay	7 (5−9)	7.6 (6−9)	6.3 (4−7)	.012
Events				
Mortality at 1 month, *n* (%)	2 (5.3)	2 (8.7)	0 (0)	.241
Composite of death or readmission at 1 month, *n* (%)	7 (15.9)	4 (16.8)	3 (15.0)	.985

Abbreviations: ACE, angiotensin‐converting enzyme inhibitors; ALT, alanine transaminase; ARB, angiotensin receptor blockers; ARNI, angiotensin receptor‐neprilysin inhibitor; AST, aspartate transaminase; BNP, brain natriuretic peptide; CA125, carbohydrate antigen 125; COPD, chronic obstructive pulmonary disease; DBP, diastolic blood pressure; eGFR, estimated glomerular filtration rate, assessed with the MDRD equation; LA, left atrial; LVEF, left ventricular ejection fraction; SBP, systolic blood pressure.

^a^ Values are presented as median interquartile range (Q_1_−Q_3_), or number (%) when indicated.

### 3.2 Loop diuretic treatment

The group treated with spironolactone showed a statistical trend to receive greater doses of i.v. furosemide (Table 1).

### 3.3 Length of stay

**Figure 2:**
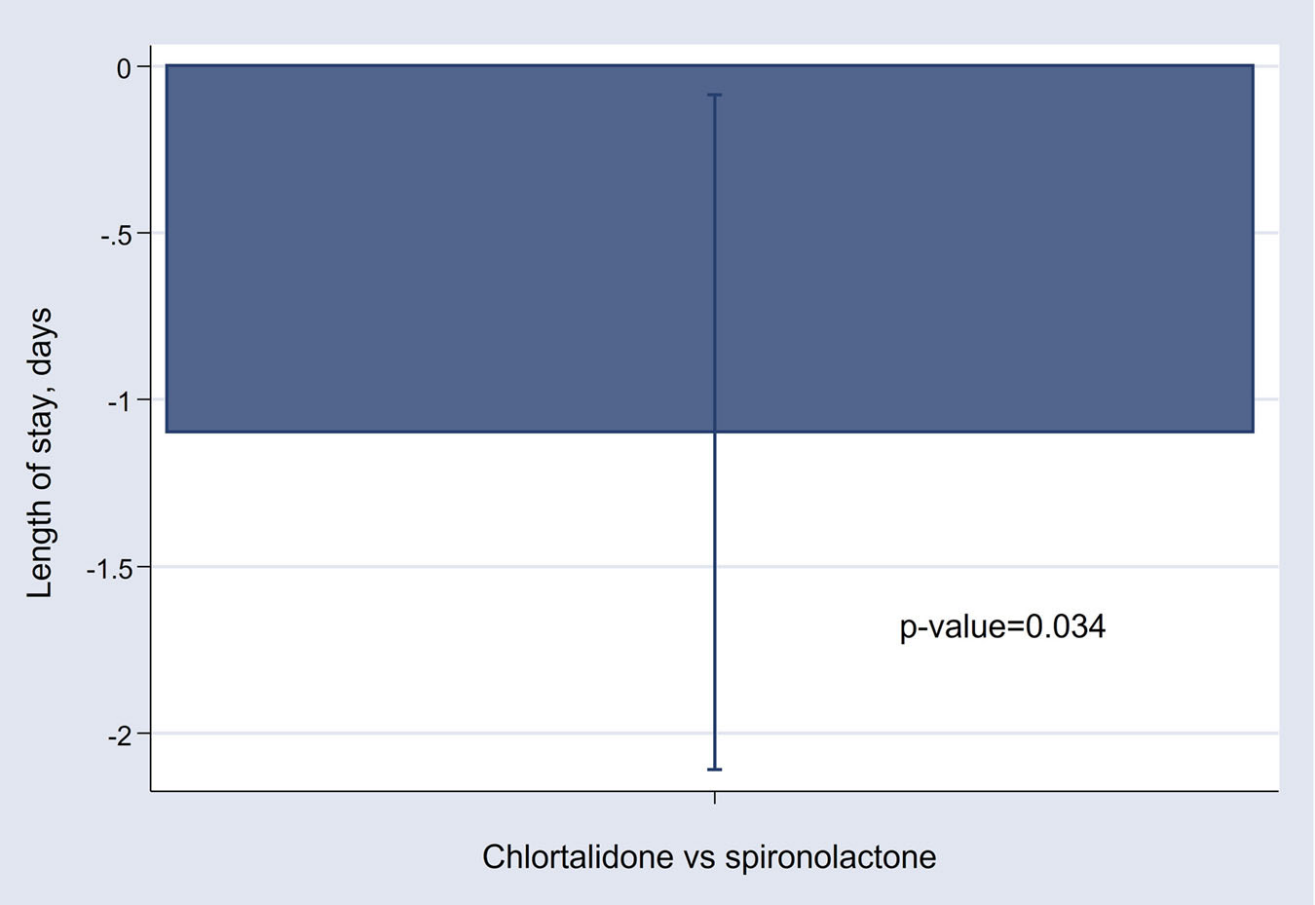
Comparison of length of stay among diuretic strategies (chlorthalidone vs. spironolactone).

The median length of stay was 7 (p25 5 to 9 p75) days. Patients treated with chlorthalidone had lower median length than those on spironolactone (6.3 [p25 4 to 7 p75] vs. 7.6 [p25 6 to 9 p75] days, *p* = .012). In a multiple linear regression model the independent predictors of length of stay were sex (*β*‐coefficient = −1.80, *p* = .002), furosemide dose (*β*‐coefficient = 0.004, *p* = .002), and chlorthalidone (*β*‐coefficient = −1.10, *p* = .034) (Figure 2).

### 3.4 Utility of spot urinary potassium/creatinine ratio for tailoring the type of diuretic regimen

Among patients with k/Cr below the median (<0.56), those on chlorthalidone showed higher natriuresis at 24 h (77 [46−92] vs. 25 [21.5‐51.5] mmol/L, *p* = .045), but not at 72 h (50 [19.5−72] vs. 40 [35−72] mmol/L, *p* = .318). In patients above the median of K/Cr (≥0.56), chlorthalidone led to a significant higher natriuresis at both 24 h (82 [52−129] vs. 63 [26−73] mmol/L, *p* = .049) and 72 h (73 [35−107] vs. 38 [19−55] mmol/L, *p* = .033). The use of chlorthalidone resulted in higher cumulative diuresis at 72 h across both ranges of K/Cr levels (4150 [3550−8000] vs. 3200 [2450−4300], *p* = .018 for K/Cr <0.56, and 6250 [5050−8000] vs. 4875 [3275−5550], *p* = .016, for K/Cr ≥0.56).

**Figure 3:**
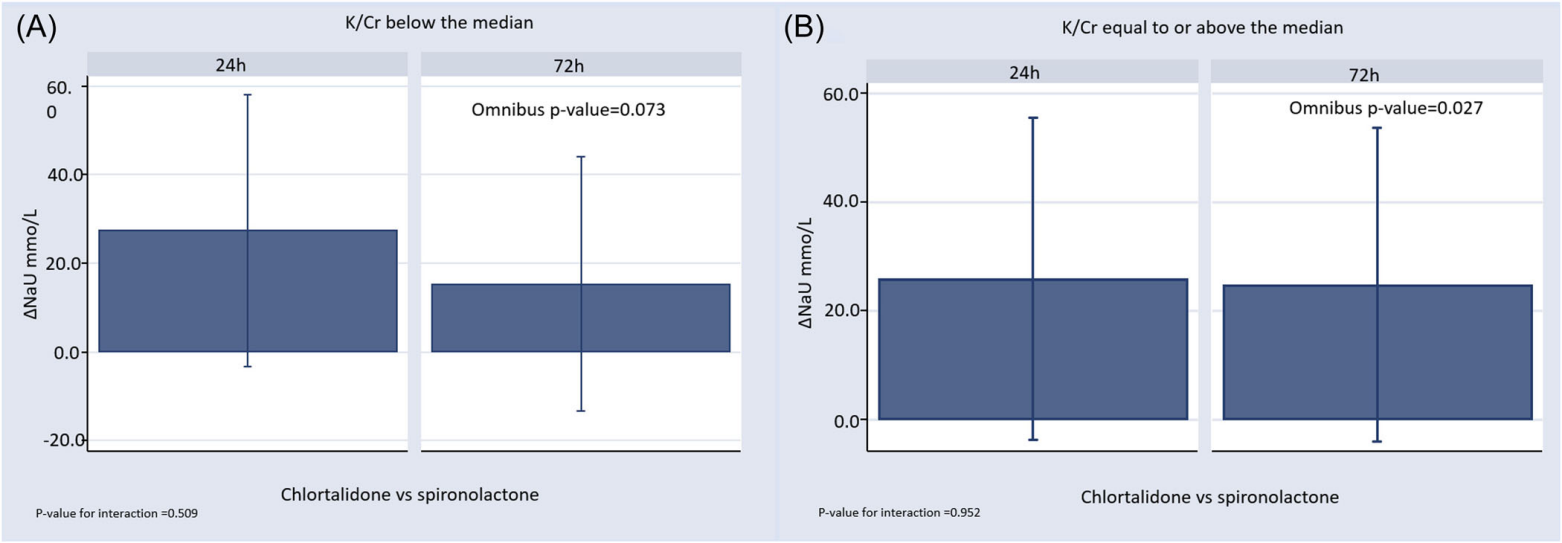
(A) Comparison of natriuresis among diuretic strategies (chlorthalidone vs. spironolactone) for K/Cr below the median (<0.56). Estimates (least squares means [LSM] with 95% confidence intervals [CI]) derived from linear mixed models for changes in natriuresis according to treatment during the 72 h follow‐up period, including the treatment x visit interaction term (24 and 72 h visits), and adjusted for urinary sodium (uNa) at baseline, age, total dose of furosemide, and baseline glomerular filtration rate. The upper section of the bar charts represents the LSM value, and the upper and lower limits of the lines represents the corresponding 95% CI. (B) Comparison of natriuresis among diuretic strategies (chlorthalidone vs. spironolactone) for K/Cr equal to or above the median (≥0.56). Estimates (LSM with 95% CI) derived from linear mixed models for changes in natriuresis according to treatment during the 72 h follow‐up period, including the treatment x visit interaction term (24 and 72 h visits), and adjusted for uNa at baseline, age, total dose of furosemide, and baseline glomerular filtration rate. The upper section of the bar charts represents the LSM value, and the upper and lower limits of the lines represents the corresponding 95% CI. K/Cr, urinary potassium to creatinine ratio.

The inferential multivariate analysis suggested a stronger natriuretic effect of chlorthalidone across k/Cr levels. Compared to spironolactone, in patients below the median of K/Cr, the use of chlorthalidone showed higher, though nonsignificant, natriuresis at 24 and 72 h (chlorthalidone vs. spironolactone: ΔuNa at 24 h: 27.5 mmo/L, 95% CI: −3.2 to 58.1, *p* = .088; and ΔuNa at 72 h: 15.4 mmo/L, 95% CI: −13.3 to 44.0, *p* = .408; omnibus *p*‐value: .073), as shown in Figure 3. Likewise, in the upper category, chlorthalidone translated into a statistically significant increase in natriuresis at 24 and 72 h (chlorthalidone vs. spironolactone: ΔuNa at 24 h: 25.7 mmo/L, 95% CI: [−3.7 to 55.4], *p* = .098; and ΔuNa at 72 h: 24.8 mmo/L, 95% CI: [−4 to 53.6], *p* = .106; omnibus *p*‐value .027), as shown in Figure 3. Estimates (*β*‐coefficients) of the covariates included in the final model for natriuresis are shown in Table 2.

**Table 2:** Estimates (*β*‐coefficients) of the covariates included in the final model for natriuresis and diuresis at 72 h.

Variable	*β*‐coefficients	CI	*p*
Estimates (*β*‐coefficients) of the covariates included in the final model for natriuresis			
Urinary sodium	0.55	0.3−0.79	<.001
Age	−0.56	−1.69 to 0.56	.328
K/Cr	−24.17	−42.24 to −6.1	.009
eGFR	0.15	−0.18 to 0.47	.371
Total dose furosemide	0.03	−1.69 to 0.56	.328
Estimates (*β*‐coefficients) of the covariates included in the final model for diuresis			
Urinary sodium	10.71	−7.47 to 28.89	.244
Age	−93.98	−1.8 to 5.08	.036
K/Cr	341.44	−1037.19 to 1720	.623
eGFR	1.51	−22.26 to 25.29	.899
Total dose furosemide	1.64	−1.8 to 5.08	.346

Abbreviations: eGFR, estimated glomerular filtrate rate; K/Cr, urinary potassium to creatinine ratio.

Multivariate analyses revealed a significant increase in 72 h cumulative diuresis irrespective of K/Cr status in those on chlorthalidone (lower K/Cr: *β*‐coefficient = 1567, 95% CI: [193−2941], *p* = .026; and upper K/Cr: *β*‐coefficient = 1936, 95% CI: [575−3297], *p* = .006). Estimates (*β*‐coefficients) of the covariates included in the final model for 72 h cumulative diuresis are shown in Table 2.

## 4 DISCUSSION

To our knowledge, this is the first study investigating the role of K/Cr to guide the choice of a second diuretic to add to furosemide in patients with AHF and suboptimal loop diuretic response. In patients with K/Cr above the median (≥0.56), both diuresis and natriuresis were higher when using chlorthalidone compared to spironolactone. After multivariate analysis, the effects on natriuresis with chlorthalidone compared to spironolactone approached a statistically significant increase at 24 and 72 h visits. The use of chlorthalidone resulted in higher cumulative diuresis at 72 h at both K/Cr levels. Of note, these results contradict our initial hypothesis, as we speculated that patients with elevated K/Cr would present greater diuresis and natriuresis with MRAs.

The hyperaldosteronism suspected to be associated with a higher concentration of potassium in urine, could thus benefit from its inhibition with MRAs has not been corroborated. In this regard, renal potassium excretion is conditioned by numerous factors,^14^, ^15^ being a particularly complex regulation. Renal potassium excretion depends: first, on the concentration of potassium in the blood, which in turn depends largely on the intake; second, on the distal sodium supply and on tubular flow; third, on the concentration of aldosterone in plasma, which, in turn, depends mainly on the concentration of potassium in the blood and angiotensin II. Therefore, we cannot assert that urine potassium levels in patients with heart failure are primarily due to secondary hyperaldosteronism, but rather depend on many determinants that prevent explicitly giving hyperaldosteronism a leading role. Also, to the best of our knowledge, there is no prior evidence showing robust evidence endorsing the association between spot urinary potassium and aldosterone activity in patients with advanced HF. The association of urinary potassium secretion and plasma aldosterone has been controversial for many years. Most of the evidence comes from animal studies. The role of aldosterone in isolation, independently, with the regulation of potassium excretion seems to be of little relevance. It is more important when plasma potassium levels increase coincident with increased intake.^15^

We speculate that patients with an elevated urine K/Cr ratio might respond better to thiazides due to better renal flow. Considering the determinants of potassium excretion in urine, potassium intake was similar throughout the whole cohort since we did not educate our patients on a restriction in potassium intake. In our cohort, we did not measure plasma aldosterone levels, since 2/3 of the patients received drugs that were inhibitors of the renin angiotensin aldosterone system, with no differences between the two groups. Patients treated with thiazides had higher systolic pressures, both at the baseline visit and at 24 and 72 h, compared to those treated with spironolactone, translating into better renal flow, which is undoubtedly a determining factor of the correct action of diuretics.

In addition, in a recent published study with the same cohort of patients, the ICARPo study,^12^ in which chlorthalidone was compared with spironolactone in terms of diuresis and natriuresis, after a multivariate analysis including K/Cr among the covariables, we observed a greater diuresis and natriuresis with chlorthalidone both at 24 and 72 h.^12^ This was the first study in the literature that compared the natriuretic and diuretic effect of chlorthalidone versus spironolactone in AHF.

It should be noted that the current ESC guidelines,^16^ as opposed to previous ones,^17^ do not contemplate the possibility of adding spironolactone as a second diuretic, and they do consider thiazide, acetazolamide, or metolazone useful as additional diuretic. Current findings support current guidelines adding new information about the utility of certain novel parameters for tailoring the diuretic strategy in patients with AHF and suboptimal loop diuretic response.

### 4.1 Study limitations

Several limitations in this study should be noted. First, this is a small single‐center observational study that only included patients with AHF and LVEF ≥50%. Second, it should be noted that urine potassium values were relatively low in the entire sample, probably due to the advanced disease and high resistance to diuretics. Third, in the current work we did not measure aldosterone levels or other surrogates of aldosterone activity. Thus, we could not examine the role of urinary potassium as a proxy of aldosterone activity. Finally, it is a small prospective single‐center observational study, in which the risk of significant residual confounding cannot be ruled out.

## 5 CONCLUSIONS

In patients with an episode of AHF‐pEF and suboptimal loop diuretic response, both diuresis and natriuresis are higher with the administration of chlorthalidone compared to spironolactone, irrespective of K/Cr status.

## CONFLICT OF INTEREST STATEMENT

The authors declare no conflict of interest.

## Data Availability

The data sets generated during and/or analyzed during the current study are not publicly available due to their containing information that could compromise the privacy of research participants but are available from the corresponding author on reasonable request.

## References

[1] Martens P , Nijst P , Mullens W . Current approach to decongestive therapy in acute heart failure. Curr Heart Fail Rep. 2015;12:367‐378. 26486631 10.1007/s11897-015-0273-5

[2] Jentzer JC , DeWald TA , Hernandez AF . Combination of loop diuretics with thiazide‐type diuretics in heart failure. JACC. 2010;56:1527‐1534. 21029871 10.1016/j.jacc.2010.06.034

[3] Mullens W , Damman K , Harjola VP , et al. The use of diuretics in heart failure with congestion—a position statement from the Heart Failure Association of the European Society of Cardiology. Eur J Heart Fail. 2019;21:137‐155. 30600580 10.1002/ejhf.1369

[4] Gottlieb SS , Khatta M , Wentworth D , Roffman D , Fisher ML , Kramer WG . The effects of diuresis on the pharmacokinetics of the loop diuretics furosemide and torsemide in patients with heart failure. Am J Med. 1998;104:533‐538. 9674715 10.1016/s0002-9343(98)00111-9

[5] Ellison DH . Diuretic therapy and resistance in congestive heart failure. Cardiology. 2001;96:132‐143. 11805380 10.1159/000047397

[6] Sigurd B , Olesen KH , Wennevold A . The supra‐additive natriuretic effect addition of bendroflumethiazide and bumetanide in congestive heart failure. Am Heart J. 1975;89:163‐170. 1090132 10.1016/0002-8703(75)90041-1

[7] Dormans TPJ , Gerlag PGG . Combination of high‐dose furosemide and hydrochlorothiazide in the treatment of refractory congestive heart failure. Eur Heart J. 1996;17:1867‐1874. 8960430 10.1093/oxfordjournals.eurheartj.a014805

[8] Kapelios CJ , Bonou M , Vogiatzi P , et al. Association between high‐dose spironolactone and decongestion in patients with acute heart failure: an observational retrospective study. Am J Cardiovasc Drugs. 2018;18:415‐422. 29971596 10.1007/s40256-018-0290-3

[9] Hensen J , Abraham WT , Dürr JA , Schrier RW . Aldosterone in congestive heart failure: analysis of determinants and role in sodium retention. Am J Nephrol. 1991;11:441‐446. 1840232 10.1159/000168356

[10] Eng M , Bansal S . Use of natriuretic‐doses of spironolactone for treatment of loop diuretic resistant acute decompensated heart failure. Int J Cardiol. 2014;170:e68‐e69. 24268982 10.1016/j.ijcard.2013.11.023

[11] Butler J , Anstrom KJ , Felker GM , et al. Efficacy and safety of spironolactone in acute heart failure: the ATHENA‐HF randomized clinical trial. JAMA Cardiol. 2017;2:950‐958. 28700781 10.1001/jamacardio.2017.2198 PMC5675712

[12] Llàcer P , Núñez J , García M , et al. Comparison of chlorthalidone and spironolactone as additional diuretic therapy in patients with acute heart failure and preserved ejection fraction. Eur Heart J Acute Cardiovasc Care. 2022;11(4):350‐355. 35167653 10.1093/ehjacc/zuac006

[13] Núñez J , Espriella R , Miñana G , et al. Antigen carbohydrate 125 as a biomarker in heart failure: a narrative review. Eur J Heart Fail. 2021;23(9):1445‐1457. 34241936 10.1002/ejhf.2295

[14] Young DB . Analysis of long‐term potassium regulation. Endocr Rev. 1985;6:24‐44. 3884328 10.1210/edrv-6-1-24

[15] Welling PA . Regulation of renal potassium secretion: molecular mechanisms. Sem Nephrol. 2013;33:215‐228. 10.1016/j.semnephrol.2013.04.002 23953799

[16] McDonagh TA , Metra M , Adamo M , et al. 2021 ESC guidelines for the diagnosis and treatment of acute and chronic heart failure. Eur Heart J. 2021;42:3599‐3726. 34447992 10.1093/eurheartj/ehab368

[17] Ponikowski P , Voors AA , Anker SD , et al. ESC Scientific Document Group . 2016 ESC guidelines for the diagnosis and treatment of acute and chronic heart failure: the task force for the diagnosis and treatment of acute and chronic heart failure of the European Society of Cardiology (ESC) developed with the special contribution of the Heart Failure Association (HFA) of the ESC. Eur Heart J. 2016;37:2129‐2200. 27206819 10.1093/eurheartj/ehw128

